# An Investigation of the Structural Strength of Transtibial Sockets Fabricated Using Conventional Methods and Rapid Prototyping Techniques

**DOI:** 10.33137/cpoj.v2i1.31008

**Published:** 2019-04-18

**Authors:** B Pousett, A Lizcano, S.U. Raschke

**Affiliations:** 1 Barber Prosthetics Clinic, Vancouver, British Colombia, Canada.; 2 Biomedical Engineering Department, Universidad Iberoamericana, Ciudad de Mexico, Mexico.; 3 MAKE + Applied Research, Centre for Applied Research & Innovation (CARI), Burnaby, British Columbia, Canada.

**Keywords:** Prostheses, Rapid Prototyping, Prosthesis Design, 3D Printing, Three Dimensional Printing, Transtibial, Socket Strength, Transtibial Socket, Thermoplastic, Lamination, Rapid Additive Manufacturing, Lower-limb Prostheses

## Abstract

**BACKGROUND:**

Rapid Prototyping is becoming an accessible manufacturing method but before clinical adoption can occur, the safety of treatments needs to be established. Previous studies have evaluated the static strength of traditional sockets using ultimate strength testing protocols outlined by the International Organization for Standardization (ISO).

**OBJECTIVE:**

To carry out a pilot test in which 3D printed sockets will be compared to traditionally fabricated sockets, by applying a static ultimate strength test.

**METHODOLOGY:**

36 sockets were made from a mold of a transtibial socket shape,18 for cushion liners with a distal socket attachment block and 18 for locking liners with a distal 4-hole pattern. Of the 18 sockets, 6 were thermoplastic, 6 laminated composites & 6 3D printed Polylactic Acid. Sockets were aligned in standard bench alignment and placed in a testing jig that applied forces simulating individuals of different weight putting force through the socket both early and late in the stance phase. Ultimate strength tests were conducted in these conditions. If a setup passed the ultimate strength test, load was applied until failure.

**FINDINGS:**

All sockets made for cushion liners passed the strength tests, however failure levels and methods varied. For early stance, thermoplastic sockets yielded, laminated sockets cracked posteriorly, and 3D printed socket broke circumferentially. For late stance, 2/3 of the sockets failed at the pylon. Sockets made for locking liners passed the ultimate strength tests early in stance phase, however, none of the sockets passed for forces late in stance phase, all broke around the lock mechanism.

**CONCLUSION:**

Thermoplastic, laminated and 3D printed sockets made for cushion liners passed the ultimate strength test protocol outlined by the ISO for forces applied statically in gait. This provides initial evidence that 3D printed sockets are statically safe to use on patients and quantifies the static strength of laminated and thermoplastic sockets. However, all set-ups of sockets made for locking liners failed at terminal stance. While further work is needed, this suggests that the distal reinforcement for thermoplastic, laminated and 3D printed sockets with distal cylindrical locks may need to be reconsidered.

## INTRODUCTION

A prosthetic socket is the interface connecting a person’s limb to the prosthetic components they use to interact with the environment. Typically, sockets are manufactured from a plaster mold of a person’s limb which is modified to create an optimized shape.^1^ The socket is fabricated over the mold using materials including thermoplastics and laminated composites. 3D scanning systems are an alternate method to digitize the patient’s limb and modify the shape.^1^ Often, the optimized shape is milled by a computer numerically controlled (CNC) milling machine and the socket is fabricated using traditional methods.^1^

As technology advances, the question emerges: ‘Is this hybrid combination of digital scanning and design technology with traditional manufacturing methods the best approach?’. Rapid Prototyping (RP) offers a time efficient way of turning the digital design into a physical socket. RP involves sectioning the digital 3D socket design into thin slices, and sending it to a 3D printer that builds the shape layer by layer.^1^ Over the past three decades, several groups have begun to create prosthetic sockets using rapid prototyping techniques.^2,3,4^

The Prosthetist is responsible for choosing fabrication techniques that provide adequate strength and safety to their patients while maximizing function.^5^ Currently, their decisions are not grounded on an evidence-based foundation as minimal evidence is available.

Furthermore, the evaluation of prosthetic sockets is not subject to any specific standard. ISO 10328: Prosthetics–Structural testing of lower-limb prostheses is the test standard that is most commonly used to test prosthetic sockets.^5^ ISO 10328 includes both static and cyclic strength tests applied in two different loading conditions, Condition I: instant of maximum loading occurring early in the stance phase of walking, and Condition II: instant of maximum loading occurring late in the stance phase of walking, for three different weight limits, P3 body mass below 60 kg, P4 body mass below 80 kg, and P5 body mass above 100 kg.^6^

Previously, this standard has been used in to evaluate the strengths of different socket attachment methods as this is frequently the point of failure in transtibial prostheses.^5^ Current, Kogleg & Barth^7^ applied the static portion of the standard and tested 10 transtibial sockets for P5 at Condition II. They compared five reinforcement materials and two resins using a 4-hole distal attachment system and found that all 10 sockets failed the ISO 10328 standard, breaking at the attachment plate.^7^ Graebner & Current^5^ investigated the strength of different socket attachment methods for composite sockets by applying the same portion of the standard as above. They found most attachment methods tested passed that aspect of the standard, especially when carbon reinforcement was used.^5^ Finally, MacKinnon^8^ used the same jig to perform the same test on three different socket attachment methods for thermoplastic sockets. He found two of the methods passed that portion of the standard when reinforced with fiberglass cast.^8^

Gerschutz et al.^9^ took a different approach and applied the static part of the ISO Standard 10328 to evaluate sockets made in a variety of facilities. For forces applied at Condition II for P6 (a mass being further above 100 kg), they found most check sockets and definitive laminated sockets and all copolymer sockets failed the standard.^9^ These studies show that there is a lot of variability in sockets fabrication techniques and attachment methods that result in sockets passing or failing this portion of the standard.

The goal of this project was to evaluate how 3D printed sockets compare to traditionally fabricated sockets made out of thermoplastics and laminated composites. This was done by applying the static portion of the ISO 10328 standard for a variety of weight limits and load both early and late in the stance phase following the same testing protocol as these previous authors to allow for comparison.^5, 7, 8^

## METHODOLOGY

### Socket Fabrication & Alignment

**FIGURE 1:**
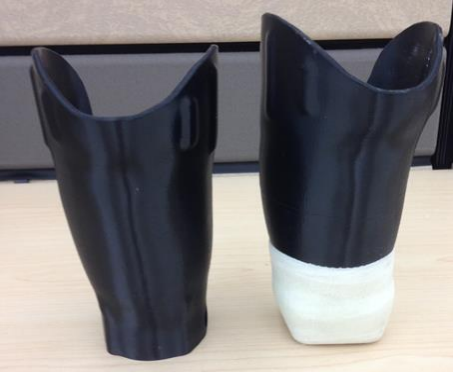
The two types of total surface bearing sockets used; the left one was made for use with locking liners with a 4-hole pattern lock distally and the right one was designed for use with cushion liners and was attached to a distal attachment block and reinforced with fiberglass wrap.

**FIGURE 2:**
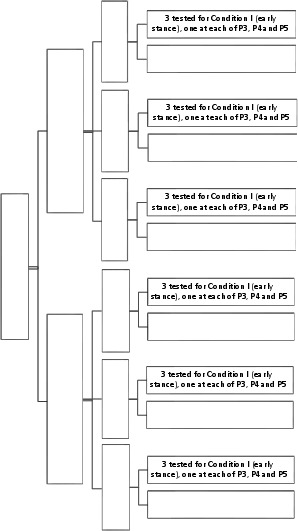
An outline of all the sockets that were fabricated for this study, which mold and materials they were made from and which conditions they were tested for.

This study chose to evaluate the strength of two different types of total surface bearing sockets, those made for cushion liners attached to a 5R1 block (“cushion sockets”) and those made for a locking liner attached distally via a 4-hole pattern lock (“locking sockets”) (FIGURE 1). It tested three different fabrication materials, thermoplastic, laminated composited and 3D printed Polylactic Acid (PLA). A total of 36 sockets were tested. See FIGURE 2 for more information.

The structural test model was manufactured from a cast of an 80 kg male with a unilateral transtibial amputation that had been modified by an experienced Prosthetist using common methods. This model was chosen as it was generic total surface bearing shape that was slightly conical, allowing multiple sockets to be removed without damaging the mold. It was slightly smaller than the average transtibial socket fit at the clinic, but it fit within the build height of the printer and it was feasible to print sockets within a reasonable timeframe of 8-9 hours. The socket is 15 cm from the patella tendon to the distal end and 32 cm in circumference around the patella tendon. One physical mold was fabricated identical to the modified cast while the other physical mold had the Fillauer cylindrical lock dummy (Chattanooga, United States) shape incorporated into the bottom of the shape. Each mold was digitized using a Spectra Scanner (Vorum, Vancouver, Canada) and converted to a 3D print file by Additive O&P (Charlotte, United States).

18 identically shaped sockets were fabricated from each of the models; 6 out of each different type of material using an identical process for each material type. All sockets were fabricated at Barber Prosthetics Clinic by a Registered Prosthetic Technician. See TABLE 1 for detailed fabrication information.

**TABLE 1.** Processes used to produce sockets. Every effort was made to ensure an identical process was followed for each socket of the same material.

Method	Thermoplastic	Laminated Composite	3D Printed
Material	12 mm Orfitrans Stiff (a transparent, rigid and thermoformable Styrene Co-Polyester)	½ oz. Dacron Felt, Nyglass, Carbon Cloth, & Resin	PLA
Process for Cushion Sockets	Blister-formed socket Attached 5R1 block using OttoBock Sealing Resin and reinforced it with 3” Scotchcast™ circumferencial wrap.	Laminated 1st stage*1, attached 5R1 block using OttoBock Sealing Resin, laminated 2nd stage*2	Print socket using fused Deposition Modelling on Rockstock Max V3 Printer. Attach 5R1 block using OttoBock Sealing Resin and reinforced it with 3” Scotchcast™ circumferencial wrap.
Process for Locking Sockets	Set lock dummy in standard alignment Blister-formed socket Drilled 4 holes distally for lock installation	Completed 1 stage lamination*3 Drilled 4 holes distally for lock installation	Print socket using fused Deposition Modelling on Rockstock Max V3 Printer with lock dummy included distally.

*1 Layup: 1 layer 1/2oz dacron felt, 2 layers nyglass, 1 layer carbon cloth from proximal trimline to 1½” distal to posterior brim trim line, 2 layers nyglass.

*2 Layup: 1 layer nyglass, 1 layer carbon clock from proximal socket trimline to 1½” distal to posterior brim trimline and from 2” proximal to the distal end to the distal end of the block, 3 layers of nyglass.

*3 1 layer 1/2oz darcron felt, 2 layers nyglass, 1 layer carbon cloth from proximal socket trimline to 1½” distal to posterior brim trimline and from 2” proximal to the distal end to the distal end of the socket, 2 x nyglass, 1 layer carbon cloth (as described above), 4 layers nyglass.

The sockets were identically aligned in a Vertical Alignment Jig (Hosmer, Fillauer, Chattanooga, United States) using the model patient’s alignment, which was 5 degrees of flexion and 2 degrees of abduction. This alignment was done similarly to previous studies, which do not follow the ISO 10328 recommendation that the alignment be set in the “worst condition”.^5,6^ This decision was made to standardize the process using a realistic alignment for the chosen model shape as this bench alignment is repeatable whereas the specifics of what makes a worse case condition is unspecified and is inconsistent with previous studies. The alignment chosen will allow future tests to be compared to the socket test done in this study. A 5R1 attachment block was used for sockets made for cushion liners, as it has been reported to be the most commonly used socket attachment methods in Canada.^8^ The sockets for the locking liners were attached using the distal 4-hole pattern in the lock mechanism. All sockets were then attached to an Ottobock (Duderstadt, Germany) titanium pyramid (5R54), a 23.2 cm aluminum pylon with a titanium connector (2R37) and a titanium tube clamp (4R52), all torqued to manufacturer’s specifications.

### Test Step-Up

**FIGURE 3.**
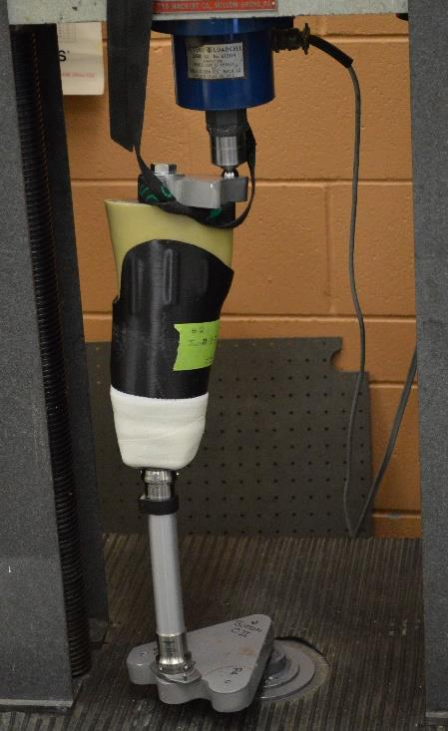
The experimental set up consisting of the socket and pylon held by a custom-made jig in the Tinius Olsen universal testing machine.

The testing was performed in a Tinius Olsen Universal Testing Machine with a 2500kg Revere Load Cell (Tinius Olsen Test Machine Co., Horsham, United States). ISO 10328 specifies the magnitude of load and where the load should be applied at the top and bottom of the set up for each condition, also called the offsets (TABLE 2).^6^ A jig was fabricated for these conditions, allowing easy and consistent setup of the socket fixture for each test done. The vertical load was applied using two 19 mm hitch balls adapted to the top and bottom lever of the Tinius Olsen universal testing machine. To evenly distribute the load through the socket a high-density urethane resin (Smooth-Cast™ 380, Smooth-On, Macungie, United States) mold of the limb was made. A steel rod was molded into the urethane to generate a better grip between the top jig and the limb mold, using a 5/8 bolt. The setup can be seen in FIGURE 3.

**TABLE 2.** The offset values for the top and bottom load application points for all conditions and levels. The forward direction is equivalent to anterior/posterior on the socket and the outward direction is equivalent to medial/lateral on the socket.

		P5		P4		P3		
**Reference plane**	Offset direction	Condition I	Condition II	Condition I	Condition II	Condition I	Condition II
**Top**	Forward Outward	82 -79	55 -40	89 -74	51 -44	81 -85	51 -49
**Bottom**	Forward Outward	-48 45	129 -19	-52 39	124 -22	-58 39	124 -23

### Test Procedure

In accordance with the load values (TABLE 3) and specifications of the structural testing of lower limb prostheses ISO 10328, all sockets were tested for a proof test and ultimate static strength test as this standard specifies.

**TABLE 3.** The static test procedure and load for each condition and level. For each condition, the setting force, proof test and ultimate static test force is applied following the protocol described below.

	P5		P4		P3	
Test procedure and test load	**Condition I**	**Condition II**	**Condition I**	**Condition II**	**Condition I**	**Condition II**
Settling test force (N)	1024	920	944	828	736	638
Proof test force (N)	2240	2013	2065	1811	1610	1395
Ultimate static test force (N)	4480	4025	4130	3623	3220	2790

For the proof test, the settling test force was applied for 30 seconds before it was removed and the set up rested at zero load for 30 seconds. The test force was then smoothly increased at a rate between 100-250 N/S to the proof test force for 30 seconds before it was removed. All load times were recorded with a stopwatch.

The ultimate strength test was conducted for all sockets that passed the proof test. Again, the settling force was applied for 30 seconds, the set up rested at zero load for 30 seconds, and the test fore was increased at a rate between 100-250 N/S to the ultimate static test force where it was maintained for 30 seconds. If the set-up had not yet failed, the load was increased until failure. Failure was the point at where the system could not support any additional load. The 10-minute wait time between the setting force and the test force specified by ISO 10328 was reduced to between 30– 60 seconds.^6^ This was done as no visible deformation or migration occurred during this period, and as this was a preliminary investigation, it allowed for more expedient testing of the samples.

### Statistical Analysis

Four independent variables were looked at: socket type (cushion sockets and locking sockets), fabrication method (thermoplastic, laminated composite and 3D printed), loading condition (Condition I and Condition II), and weight limit (P3, P4 & P5). Two dependent variables, “Proof Test Performance” and “Ultimate Strength Test Performance” each had two possible outcomes: “pass” and “fail”. Statistical analysis (SPSS-IBM, Armonk, USA) for socket type and loading condition were evaluated by Fisher’s Exact test while fabrication method and weight limit were evaluated by Chi-square test.

## RESULTS

### Cushion Sockets attached distally via a 5R1 block

**FIGURE 4.**
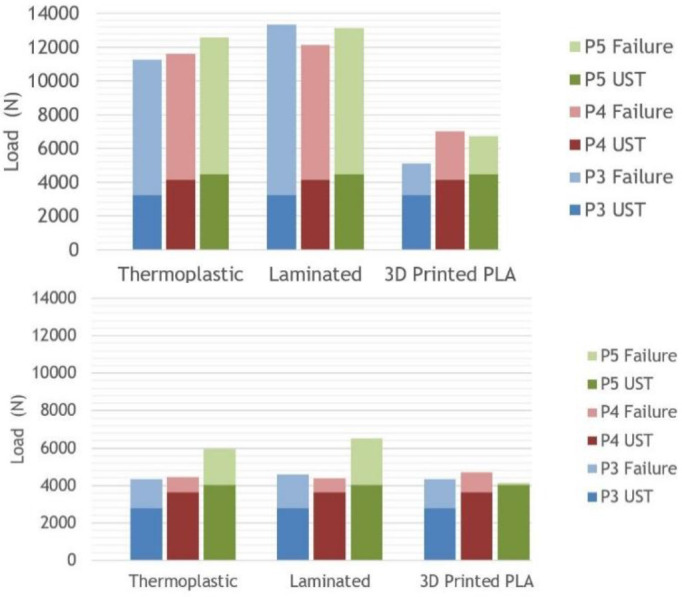
**A (Top):** For cushion sockets at Condition I (early stance phase), all set ups failed above the ultimate strength test (UST) values specified in ISO 10328; **B (Bottom):** For cushion sockets at Condition II (late stance phase), all set ups failed above the ultimate strength test values specified in ISO 10328. In 2/3 cases, the modular components were the cause of failure.

**FIGURE 5.**
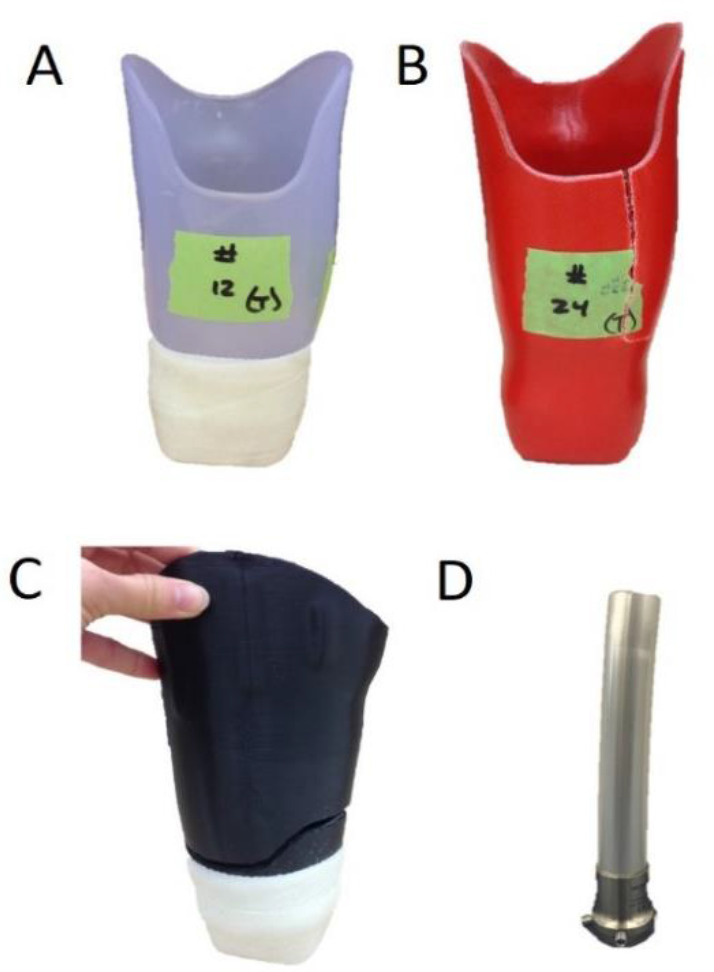
Typical failure methods for sockets made for cushion liners for the following conditions: (A) thermoplastic sockets at Condition I (B) laminated composite sockets at Condition I (C) 3D Printed PLA sockets at Condition I (D) sockets of all material types at Condition II.

All 9 sockets passed the ultimate strength test for both Condition I and II (FIGURE 4A&B), however the failure levels and methods varied. For Condition I, thermoplastic sockets yielded, laminated sockets cracked up the posterior wall and 3D printed socket broke circumferentially above the Scotchcast™ (FIGURE 5). For condition II, 2/3 set-ups for each of the materials failed because the pylon bent and yielded, often while the socket was left intact (FIGURE 5).

The strength to weight ratios were then compared for all sockets along with the failure methods (TABLE 4 A&B). For Condition I, the laminated composites had the highest strength to weight ratios, followed by the thermoplastic sockets and the 3D printed socket. The 3D printed sockets were on average approximately 75% of the weight of thermoplastic sockets and withstood approximately 71% of the force. For Condition II, the strength to weight ratio is less relevant due to the failure methods being in the modular components.

**TABLE 4.** **A:** Strength to weight ratios of cushion sockets for Condition I; **B:** Strength to weight ration of cushion sockets for Condition II.

A: Condition I					
Material	Force (N)	Socket Weight (g)	Strength to Weight	Percentile (%)	Failure
Laminated Composite	13132	429	30.61	100	Crack - posterior wall
Laminated Composite	13341	450	29.65	97	Material yield - anterior proximal gap
Laminated Composite	12113	450	26.92	88	Crack - posterior wall
Thermoplastic	12566	709	17.72	58	Material yield - anterior proximal gap
Thermoplastic	11608	738	15.73	51	Material yield - anterior proximal gap
Thermoplastic	11264	734	15.35	50	Material yield - anterior proximal gap
3D Printed PLA	7001	541	12.94	42	Circumferential break above Scotchcast™
3D Printed PLA	6725	542	12.41	41	Circumferential break above Scotchcast™
3D Printed PLA	5107	544	9.39	31	Circumferential break above Scotchcast™
**B: Condition II**					
Laminated Composite	6505	410	15.87	100	Distal attachment screw
Laminated Composite	4581	420	10.91	69	Pylon
Laminated Composite	4384	437	10.03	63	Pylon
3D Printed PLA	4707	544	8.65	55	Pylon, socket crack - posterio
Thermoplastic	5958	733	8.13	51	Attachment
3D Printed PLA	4355	547	7.96	50	Pylon, socket crack - posterior
3D Printed PLA	4143	543	7.63	48	Circumferential break above Scotchcast™
Thermoplastic	4434	733	6.05	38	Pylon
Thermoplastic	4340	736	5.90	37	Pylon

### Locking sockets attached distally via a 4-hole pattern lock

**FIGURE 6.**
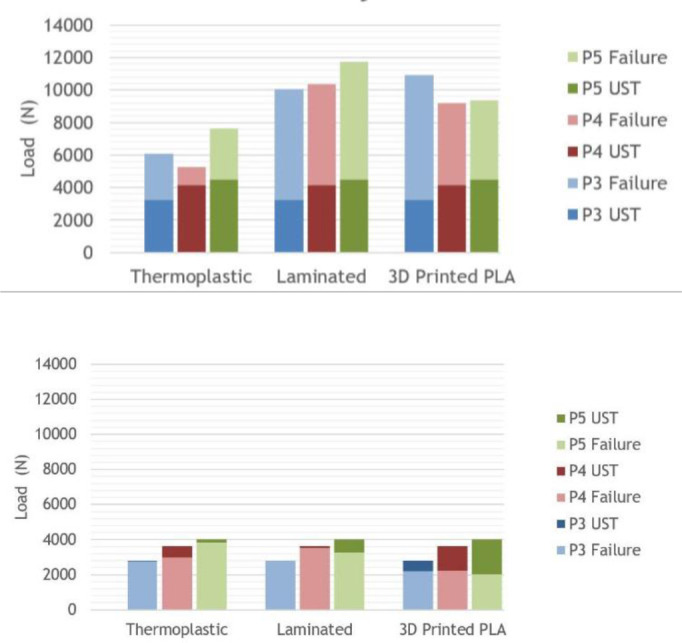
**A (TOP):** For locking sockets at Condition I (early stance phase), all of the set ups failed above the ultimate strength test values specified in ISO 10328; **B (Bottom):** For locking sockets at Condition II (late stance phase), all the set ups failed below the ultimate strength test values specified in ISO 10328.

**FIGURE 7.**
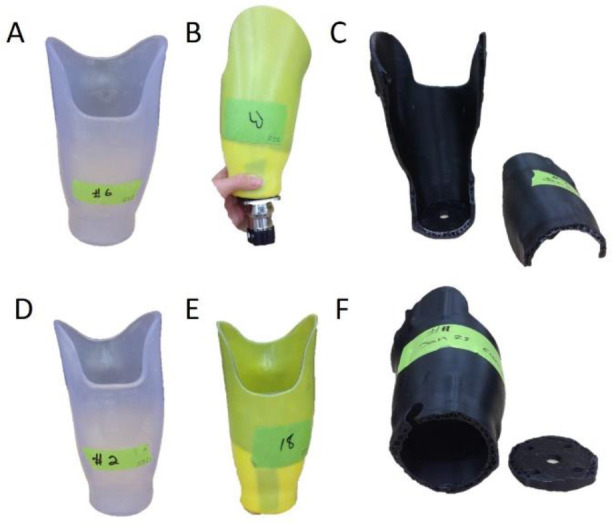
Typical failure methods for sockets made for locking liners. At Condition I: (A) thermoplastic sockets yielded and deformed, (B) all laminated composite sockets failed differently with one socket separating from the pyramid when the lock broke, and (C) all 3D Printed PLA sockets broke circumferentially at the distal end and up the sides. At Condition II: (D) thermoplastic sockets yielded and deformed and the locks broke, (E) the laminated composite sockets’ lock mechanisms broke and (F) 3D Printed PLA sockets broke circumferentially at the distal end.

All 9 sockets passed the ultimate strength tests at Condition I, however, none of the sockets passed the ultimate strength test for Condition II, and one socket didn’t pass the proof test (FIGURE 6. A&B). For Condition I, thermoplastic sockets yielded around the lock, laminated socket broke either along the posterior wall or within the lock mechanism, and the 3D Printed sockets broke circumferentially around the distal end and split up the sides (FIGURE 7). For Condition II, the thermoplastic sockets yielded around the lock, the laminated sockets’ lock mechanisms broke, and the 3D Printed sockets broke circumferentially around the distal end (FIGURE 7).

The strength to weight ratios were then compared for all sockets along with the failure methods (TABLE 5 A&B). For Condition I, the laminated composites had the highest strength to weight ratios, followed by the 3D printed sockets and the thermoplastic sockets. The 3D printed sockets weighed on average approximately 84% of the weight of thermoplastic sockets but withstood approximately 180% of the force. For Condition II the thermoplastic sockets were slightly stronger than the 3D printed sockets but none of them passed the standard.

**TABLE 5.** **A:** Strength to weight ration of locking sockets at Condition I; **B:** Strength to weight ration of locking sockets at Condition II.

A: Condition I					
Material	Force (N)	Socket Weight (g)	Strength to Weight	Percentile (%)	Failure
Laminated Composite	11730	256	45.82	100	Lock mechanism
Laminated Composite	10058	243	41.39	90	Pyramid adaptor
Laminated Composite	10364	252	41.13	90	Crack - posterior wall
3D Printed PLA	10925	368	29.69	65	Circumferential break - distal end
3D Printed PLA	9355	366	25.56	56	Circumferential break - distal end
3D Printed PLA	9197	364	25.27	55	Circumferential break - distal end
Thermoplastic	7650	446	17.15	37	Material yield - proximal anterior gap
Thermoplastic	6091	431	14.13	31	Material yield - proximal anterior gap
Thermoplastic	5241	423	12.39	27	Material yield - proximal anterior gap
**B: Condition II**					
Laminated Composite	3526	249	14.16	100	Material yield – lock broken
Laminated Composite	3278	253	12.96	91	Material yield – lock broken
Laminated Composite	2818	252	11.18	79	Material yield – lock broken
Thermoplastic	3000	426	7.04	50	Material yield around lock
Thermoplastic	2763	437	6.32	45	Material yield around lock
Thermoplastic	2853	460	6.20	44	Material yield around lock
3D Printed PLA	2243	366	6.13	43	Circumferential break around distal end
3D Printed PLA	2189	367	5.96	42	Circumferential break around distal end
3D Printed PLA	2020	365	5.53	39	Circumferential break around distal end

There were no significant differences observed between socket types at the proof test however, at the ultimate strength test, 100% of all sockets with cushion liners passed while only 50% of those with locking liners passed the test. Fisher’s Exact test found a statistically significant association between liner type and ultimate strength test, p=0.001. In looking at the strength of the association, results of a Phi test showed a strong association between liner type and ultimate strength test results, φ=0.577, p=0.001.

Similarly, there were no significant differences observed between Condition I and Condition II at the proof test however, at the ultimate strength test, 100% of sockets passed at Condition I while 50% of passed at Condition II. Results showed a statistically significant strong association between test condition and ultimate strength test results (Fisher’s Exact p=0.001; φ=0.577, p=0.001)

When comparing the ultimate strength test results based on manufacturing methods or weight classification there were no statistically significant differences, however, it is worth noting that 25% of sockets failed for each manufacturing method, all for locking liners at Condition II. Further testing may produce more decisive results.

## DISCUSSION

This study evaluates the static strength of sockets made using a variety of fabrication techniques, including 3D printing, laminated composites and thermoplastics. It employs the methodology used by previous studies to test prosthetic sockets, outlined in ISO 10328. This study expended beyond this methodology as it looked at forces in both early stance phase and late stance phase, which previous studies do not do.

## Sockets Made for Cushion Liners

### Thermoplastic Sockets:

Thermoplastic sockets are used as diagnostic sockets. The transparency of this material allows for visual inspection of the socket environment to guide the prosthetist in adjusting the socket shape. Thermoplastic sockets are heavier and have less strength than laminated composite sockets and, when tested to failure did not break catastrophically. A study conducted by Mackinnon^8^ found that thermoplastic sockets attached using resin and Scotchcast™ to a 5R1 block failure at Condition II occurred at 4792 N. These results are comparable to the current study which found thermoplastic sockets using the same attachment methods failed, on average, at 4910 N, but with the socket tested to P5 failing at 5958 N. This increased strength could be from a variety of factors such as using a different socket shape, differences in plastic thickness, or differences in the height and thickness of the reinforcement material.

### Laminated Composite Sockets:

Definitive sockets are made from laminated composites. In daily clinical practice, laminated sockets do not often break over the typical lifetime of a prosthesis. In this study, laminated composite sockets had the highest strength to weight ratio and withstood the highest force. This was especially true for Condition I (at early stance) where the sockets failed at approximately 3 times the ISO standard. This strength is dependent on many factor as discussed below.^9^ Two other studies evaluated the strength of laminated composite sockets, for Condition II for people weighing over 100 kg, using a similar experimental set up. The first study found their sockets failed between 1836 – 3160 N with the lamination failing at the pyramid attachment point.^7^ The second study found that for socket reinforced with carbon weighing between 616 – 795g, failure occurred between 4247– 5663 N.^5^ Different material lay-ups and socket attachment methods were found to increase the strength of laminated composite socket.^5,7,9^ This study also concluded that modular components began to fail above 5400 N of force.^5^

The sockets tested at Condition II in the current study, weighed between 410–450 g and broke between 4384 and 6505 N. This is approximately double the load reported by the first study and similar to results in the second study, despite sockets in this study weighing much less. Reasons for this include material selection, layer order, laminating protocol and socket attachment methods used. This is to be expected, as studies have reported a large variation in socket strength depending on who manufactures it.^9^ Findings of the second study were supported by this study which found that set-ups failed at the modular components; either because the distal attachment screw sheared or the pylon yielded. The current study indicates that for forces applied at Condition II, an average force of 4800 N resulted in failure of the modular components. Further testing is required due to the small sample size.

### 3D Printed Sockets:

3D printing technology has been identified as having the potential to benefit the production of prosthetic sockets.^3,4,10,11^ For example, in the current study, the 3D printed sockets took 9 hours and 9 minutes to print but required much less active time from a technician than traditional manufacturing methods. While 3D printing allows for rapid prototyping of custom designs, decreased manufacturing times and increased opportunities for collaboration, the main limitation continues to be the lack of standardization and regulation which may place patients at risk of receiving unsafe devices.^3,4,12^ One way to evaluate the safety of 3D printed sockets is to explore how they compare strength wise to other fabrication methods available.

For sockets made for use with cushion liners, this preliminary testing provides evidence that 3D printed sockets are strong enough statically to be used with patients as they passed the standards for all weight limits. However, when tested to failure, they failed at approximately half of the force of traditional manufacturing methods. This is hypothesized to be as a result of the material properties and manufacturing process. While traditional manufacturing methods involve either a solid sheet of plastic, which yields before it breaks, or layers of sheets of reinforcement materials, often braided or weaved for strength, 3D printing deposits material in layers, thus making it inherently weaker. When force was applied, it appeared that the 3D printed material sheared between layers. There are many factors in the printing process and design that may be able to increase the strength of these sockets such as by changing print orientation, infill pattern, adding corrugations, changing material types or using a different type of printer. Subsequent work completed by Campbell et al. provides preliminary support that for sockets made for cushion liners 10% changes in infill percentage does not affect the strength.^13^

For Condition I (early in stance phase), the sockets failed well above the ISO standard, by cracking circumferentially about the Scotchcast™ reinforcement. This indicates that the force is being concentrated there, which could be decreased using different manufacturing methods described above. For Condition II (late in stance phase), the modular components failed before the sockets failed. Modular components are regularly used in clinical practice without negative consequences. It is likely that since they are breaking before sockets are breaking, 3D sockets will survive the impact put on them statically.

Another issue raised is that 3D printed sockets break catastrophically, while other manufactured materials yield or tear more slowly. This catastrophic breaking may present dangers to patients who could be injured in this process. Additional work is required to further investigate this issue and determine if this drawback can be avoided, as well as to see how this material acts when going through cyclical testing.

Inherent in rapid prototyping is the adjustability and flexibility in the manufacturing methods – there are infinite designs, material choices and print settings that can be adjusted to influence the final product. As in conventional manufacturing methods, this variability will largely influence how strong sockets are.^9^ More work is required to evaluate these different parameters and give guidance to which choices result in better outcomes.

## Sockets Made for Locking Liners

This study presents some preliminary evidence that the use of cylindrical locks in prostheses should be re-considered. Regardless of the manufacturing methods used, the sockets with locks did not pass the ISO standard for forces applied at Condition II, and in all cases the material around the lock either yielded or cracked. Unless modifications are done to relieve the stress concentration from this point or include additional reinforcement, these sockets may fail when patients are using them. Alternatively, other lock mechanisms may be an option as they result in different distal socket shapes which may have less concentrated stress points and may withstand higher forces. This preliminary evidence supports that 3D printed sockets should not be used to create sockets with distal cylindrical locks.

### Limitations

Balancing the production of clinically-relevant and scientifically sound evidence with the feasibility of completing the research leads to several limitations which need to be addressed. First of all, sockets are not subject to ISO 10328 testing. However, as the other components in lower limb prostheses are subject to this standard and as several previous studies^5,7,9^ employed this methodology, it is reasonable to use 10328 as an evaluation tool for socket strength. The 2006 version of ISO 10328 was used as the 2016 version was not yet released at the beginning of testing.

The protocol outlined in the standard was followed as closely as possible, however several changes were made in order to allow the results to be compared to other studies and to make it feasible to conduct in a timely and cost-efficient manner. At this time point, only the static portion of the structural tests were conducted due to the length of time required to cyclical testing. However, plans are in place to continue work on cyclical testing after addressing some of the limitations uncovered through this study. When selecting a model, the standard does not specify a suitable size or shape, so a model was chosen that fulfilled technical limitations and resulted in a more expedient process. The model chosen was slightly smaller than previous studies, but it is a realistic mold, copied from a patient’s everyday prosthesis, and it was consistent across all samples. The alignment for the model was also taken from the patient’s every day alignment, which is a fairly neutral bench alignment. The standard outlines using a worse-case alignment, but as this is not precisely defined, and as all previous studies used a standard bench alignment, a standard bench alignment was used here too. This allows for comparison with other studies and consistency between samples.

Finally, due to financial, time and resource constraints, two modifications were made to the testing protocol. First, only one sample was tested for each weight limit and condition. While the standard recommends testing a minimum of two samples of each condition, choosing only one sample allowed testing at both early stance and at late stance which had not been completed in any previous study on socket strength. This resulted in new findings and directions for future research to be uncovered. Second, the wait time between the test force and the ultimate strength test force was reduced. This significantly reduced the testing time required, allowing for more samples to be tested.

Other limitations arose from the results of the socket tests. When completing the testing for Condition II, the endoskeleton modular components often failed before socket was affected. While pylons are tested to ISO 10328, these components broke prematurely and prevented the specific testing of the socket. In future studies, solid pylons can be used to isolate the force on the socket attachment and evaluate the socket strength more directly. Also, while completing testing on sockets made for locking liners, the lock mechanism frequently broke. In future studies, a lock mechanism which does not act as part of the structural attachment to the modular components may result in stronger sockets.

## Future Work

There is a need for continued work on this topic of 3D printing to support its use in prosthetic fabrication in an evidence-based and safe manner. For static strength testing, future work may include testing larger models with worse-case scenario alignment and larger sample sizes. There is also an endless combination of material choices, design options and 3D print parameters that can be explored. More specifically, the distal attachment could be strengthened, particularly in sockets made for locking liners, to extend the use of 3D printing to locking liners. Also, if design or material options could eliminate the catastrophic nature of the 3D printing failure, patient safety would be significantly enhanced and the adoption of this technology would be more widely accepted.

Beyond static testing, cyclical testing of 3D printed sockets must also be done to complete the testing palate. Until information is known on how this material performs over time, clinicians cannot be confident that this manufacturing method will meet the demands of ambulation. Future work should focus on expanding the static testing that has been done to cyclical tests in order to present a more complete picture of how this technology will work for patients.

In addition to strength, there are many other factors that can be explored including the personnel and material costs of using 3D printing over other manufacturing methods, the ease of fabrication, quality and consistency of devices fabricated, and the methods of introduction of this method into clinical practice.

## CONCLUSION

This study explored the strength of 3D printed prosthetic sockets in comparison with two other techniques that are currently used in clinical practice. It was found that all 3D printed sockets made for use with cushion liners withstood the loads specified by the ISO standard. In addition, at terminal stance, in many cases the pylons yielded before the sockets broke. As this is not routinely seen in clinical practice, it provides some evidence that the sockets are stronger than the modular components and therefore statically safe to use on patients. However, one notable limitation to the incorporation of 3D printed sockets into practice is the catastrophic nature of the failure and thus the potential serious risk it can pose to the patient. Further evaluation needs to be conducted to explore how 3D printing manufacturing methods can affect the strength of sockets and the nature of the failure.

## DECLARATION OF CONFLICTING INTERESTS

The authors have no conflicts of interest to declare.

## SOURCES OF SUPPORT

Materials and components were provided by Barber Prosthetics Clinic, OrtoPed & OttoBock.

## AUTHOR CONTRIBUTION

- **Brittany Pousett**
  Conception and design of the work. Supervision of fabrication of sockets. Data analysis and interpretation. Drafting of the manuscript.
- **Aimee Lizcano**
  Conception or design of the work. Data collection. Data analysis and interpretation. Drafting of the manuscript.
- **Silvia U Raschke**
  Canadian Supervising Academic. Conception and design of the work. Guided data analysis. Critical revision of the manuscript.
